# Acute Inflammatory Responses to Blood Flow Restriction Training: A Systematic Review

**DOI:** 10.1186/s40798-025-00926-6

**Published:** 2025-10-21

**Authors:** Sarah Barawi, Kevin Happ, Michael Behringer

**Affiliations:** Department of Sports Sciences, Goethe University Frankfurt, Frankfurt am Main, Germany

**Keywords:** Vascular occlusion training, Immune cell recruitment, Leukocyte response, Cytokine response

## Abstract

**Background:**

The effects of Blood Flow Restriction (BFR) training are well-established, but its impact on the inflammatory response remains unclear. This systematic review evaluates whether BFR training induces acute inflammation by analyzing changes in inflammatory parameters.

**Methods:**

This review was conducted according to PRISMA guidelines. The literature search was performed across PubMed, Web of Science, BISp-Surf and Google Scholar up to July 2025. Studies were included if they reported acute changes in inflammatory markers within 72 h after BFR training, as well as macrophage presence up to 14 days. Only trials involving healthy adults with inflammatory parameters assessed via peripheral blood or muscle biopsy were considered. Risk of bias was assessed using RoB 2 and ROBINS-I. Standardized mean differences (SMDs) were calculated to quantify within-study changes. In addition, relative percentage changes were calculated to enable a comparison of the magnitude of inflammatory responses across studies. An effect direction plot was created to summarize the direction of inflammatory marker changes (SWiM 2020).

**Results:**

Nine studies involving 189 healthy adults were included in the systematic review. Transient increases in total leukocytes (18–33%) and lymphocytes (37–43%) were consistently observed in peripheral blood following exercise. Significant increases in total tissue macrophages (200%) were also reported. Findings on neutrophils (up to + 40%), cytokines (up to + 340%), and lymphocyte subpopulations (TCD4⁺: +25%, TCD8⁺: +39%) varied across studies.

**Conclusion:**

The findings suggest that BFR training induces acute inflammation, characterized by transient leukocytosis, lymphocytosis, and increased macrophage activity. However, the variability in neutrophil and cytokine responses, as well as in lymphocyte subsets, may be attributed to variations in training parameters and methodological approaches. Overall, these responses appear comparable to those observed following high-load resistance training (HL-RT). Further research is needed to clarify the underlying mechanisms and their potential contribution to muscle adaptation.

**Supplementary Information:**

The online version contains supplementary material available at 10.1186/s40798-025-00926-6.

## Key Points

1. BFR training can induce acute changes in inflammatory markers, including transient increases in circulating total leukocyte and lymphocyte counts and a sustained presence of macrophages in muscle tissue. Neutrophil and cytokine responses, as well as lymphocyte subsets, showed variability across studies.
2. The magnitude of immune cell responses following BFR training appear similar to that observed after HL-RT, where immune cell recruitment has been linked to regenerative processes. These responses, particularly the sustained presence of M2 macrophages, raise the possibility that inflammation following BFR training may also contribute to muscle regeneration.
3. Further research is needed to clarify the underlying mechanisms and potential role of BFR-induced inflammation in muscle adaptation. Standardized training and measurement protocols are also required to improve comparability.

**Supplementary Information:**

The online version contains supplementary material available at 10.1186/s40798-025-00926-6.

## Introduction

Over the past decade, numerous studies have shown that combining low-load resistance training (LL-RT) (20–30% of one repetition maximum, 1-RM) with blood flow restriction (BFR) can induce muscle hypertrophy comparable to a traditional high-load resistance training (HL-RT) [1–3]. BFR is a training method that involves applying a pneumatic cuff to the proximal end of a limb, thereby reducing the arterial blood supply to the working muscles and occluding venous return [2–4]. This vascular restriction leads to local hypoxia, which promotes the accumulation of metabolites such as lactate [5–7]. It is assumed that metabolic stress and mechanical tension act synergistically, potentially activating several adaptive mechanisms [7], including early recruitment of fast-twitch muscle fibres, muscle cell swelling, and the release of anabolic hormones such as growth hormone [6–8]. These responses are thought to contribute to skeletal muscle hypertrophy and strength gains [6–8].

In this context, BFR training has been shown to improve cardiovascular parameters, enhance functional performance, and reduce pain perception [7]. These unique characteristics make BFR training particularly suitable for rehabilitation and for individuals who may not tolerate the high mechanical load of traditional HL-RT.

While the physiological adaptations to BFR training are well-established, its potential effects on inflammatory responses remain unclear. Exercise-induced inflammation has been observed following both endurance and resistance training and is believed to play a key role in muscle adaptive processes [9–16]. This response is characterized by an acute, intensity-dependent rise in leukocytes such as neutrophils, monocytes, and lymphocytes, followed by cytokine release [11–15, 17]. In exercise-induced muscle damage (EIMD), macrophages appear to infiltrate the affected tissue, where they likely play a central role in tissue repair and regeneration [11, 18–20]. The acute mobilization of immune cells typically persists for up to 72 h [10], while macrophages have been observed to remain within the tissue for up to 14 days [21–23].

Exercise-induced inflammation is believed to be driven by multiple factors, including mechanical force and metabolic stress, with contributions from hemodynamic shear stress and hormonal mediators such as catecholamines [10, 24]. During eccentric contractions in HL-RT, high mechanical shear forces lead to EIMD, characterized by sarcolemmal disruptions that trigger inflammatory responses through cytokine release and immune cell recruitment [9, 11, 13]. Although some studies have reported substantial muscle damage following BFR training [25, 26], it typically causes less damage than HL-RT [27–32]. Therefore, inflammatory responses observed after BFR training are likely driven primarily by metabolic stress and its downstream effects. Accordingly, endurance exercise has been shown to induce inflammatory responses through metabolic stress, mediated by the continuous energy demand in muscles [10, 15, 33]. This increased oxygen consumption generates reactive oxygen species (ROS), which contribute to oxidative stress [33] and are believed to activate signaling pathways that trigger inflammatory responses [34, 35]. Similarly, during BFR, the venous occlusion induced by the cuff activates mechanisms that may contribute to the inflammatory response. The reduced blood flow creates an hypoxic environment, promoting metabolic stress [36, 37]. The subsequent ischemia-reperfusion sequences increase ROS production and lead to oxidative stress, which may activate signaling pathways responsible for recruiting and mobilizing immune cells [38]. Given the overlap in intracellular stress conditions with endurance exercise, BFR training may trigger similar inflammatory pathways and thus contribute to exercise-induced inflammation. In line with this, factors such as cuff pressure and width, as well as contraction type, may affect the extent of ischemia and reperfusion, thereby modulating local shear stress. Subsequently, these parameters may influence inflammation-related signaling pathways.

Despite the previous narrative review by Rossi et al. [39] discussing the role of inflammation in muscle adaptation to BFR training, there has been no systematic analysis of the available evidence regarding acute inflammatory responses following BFR training. Therefore, this review aims to fill this gap by examining changes in inflammatory parameters as indicators of acute inflammation. Acute responses will be analyzed within the first 72 h following BFR training in healthy individuals, with consideration also given to macrophage activity, which may persist for up to 14 days. Given the potential involvement of exercise-induced inflammation in adaptive processes [9, 11–13, 15], this review may offer valuable insights for future research into the role of inflammation in muscle adaptation, which could inform the development of training and recovery approaches.

## Methods

This systematic review was conducted in accordance with the PRISMA guidelines [40] and was not registered in PROSPERO, as the work was already at an advanced stage when registration was considered. No ethical approval was required as the research was previously published.

### Eligibility Criteria

The inclusion criteria were defined according to the PICOS framework [40]:

- Population (P): Healthy participants.
- Intervention (I): BFR in combination with resistance or endurance training.
- Comparator (C): Studies with or without comparator groups.
- Outcome (O): Studies reporting changes in acute inflammatory parameters (e.g. leukocytes, cytokines, macrophages) measured pre- and post-intervention (≤ 72 h, or ≤ 14 days for macrophages).
- Study design (S): Pre–post intervention studies (randomized and non-randomized trials) were included to provide a comprehensive overview of the current evidence.

Studies were excluded if they:

- were not published in English or were not peer-reviewed.
- focused solely on pleiotropic cytokines, including myokines (e.g., IL-6), as their isolated measurement does not allow for sufficient attribution to inflammatory processes.
- involved concurrent training protocols, which did not allow separate evaluation of inflammatory effects of endurance or resistance training.
- were published as case reports, pilot studies, reviews, editorials.

### Information Sources

Studies were identified by searching the following electronic databases: PubMED/MEDLINE, Web of Science, Federal Institute of Sports Science Germany (BISp SURF) and Google Scholar. The reference lists of included studies were also screened to identify potentially relevant studies. The final search of all databases was conducted in July 2025. All studies published after this date or before 1 January 1998, were excluded from the systematic review.

### Search Strategy

To identify specific phases following BFR training that could be related to an inflammatory process, two independent reviewers conducted the search using predefined criteria. In PubMed, both Medical Subject Headings (MeSH) and text word tags (tw) were applied to ensure comprehensive coverage of indexed and non-indexed records. Truncation symbols (*) were applied to capture word stem variations. In Web of Science, the same terms were entered as free-text searches across title, abstract, author keywords, and Keywords Plus. In Google Scholar and BISp SURF, a structured free-text search was performed. Search terms related to BFR training and inflammatory responses were combined using Boolean operators (AND/OR). The detailed search strategy for each database is provided in Additional file (Table S1).

### Study Selection

A structured screening procedure was implemented to identify and select eligible studies, with Rayyan (http://rayyan.qcri.org) used to organize and evaluate records throughout the review process [41].

Studies were selected by screening the titles and abstracts according to predefined eligibility criteria. After reviewing the abstracts of articles that appeared potentially relevant or raised questions, the full texts were carefully reviewed and analyzed. Two independent reviewers conducted the selection process to ensure the reliability and consistency of the review. In cases of disagreement, consensus would have been reached through discussion or consultation with a third reviewer. However, no discrepancies occurred during the screening or selection process.

### Data Items and Extraction

The following information was extracted for the included studies: year of publication, authors’ names, participant descriptions (including sample size, gender distribution, age, height, weight and fitness level) training protocol (workload intensity, trained muscle groups, sets and repetitions per session, rest periods and cuff pressure, cuff width, contraction type), as well as measurement time points of blood samples or biopsy collection. Additionally, data on acute inflammatory parameters (e.g., leukocytes, including lymphocytes, neutrophils and macrophages as well as cytokines) following BFR training were extracted.

### Data Analysis

Relative percentage changes in inflammatory markers were calculated based on data points extracted from graphical data using the PlotDigitizer software (https://plotdigitizer.com), which has been shown to be a valid and reliable tool [42]. Axes were calibrated based on the original figure scales, and values were extracted to descriptively compare the magnitude of changes in inflammatory markers following BFR training across studies.

As meta-analysis was not feasible due to heterogeneity of protocols and outcomes, a narrative synthesis was performed. In accordance with SWiM (Synthesis Without Meta-analysis) 2020 guidelines [43], an effect direction plot was created to summarize the direction and consistency of changes across immune parameters (see Table 3).

Standardized mean differences (SMDs) and 95% confidence intervals were calculated to quantify between-group differences within studies (e.g., BFR vs. control) [44]. The SMD was calculated as the difference between the group means divided by the pooled standard deviation, with Hedges’ g correction applied for small sample sizes. These calculations were performed using the validated online tool Psychometrica [45].

### Quality Assessment of Studies

The quality of included randomized trials was assessed using the “risk-of-bias tool for randomized trials 2” (RoB 2, 2019 Version), which categorizes the risk of bias as low, some concerns or high [46]. For crossover trial, the same RoB 2 tool was used, with the addition of an extra dimension (Domain 1b). As this domain applies only to crossover designs, it was not assessed for the parallel-group trials and is shown in grey in the traffic-light figure to indicate non-applicability. For non-randomized studies, the ROBINS-I (Risk of Bias in Non-randomized Studies of Interventions) tool was utilized to assess the risk of bias in the included intervention studies [47]. The ROBINS-I tool classifies the risk of bias as low, moderate, serious, or critical.

## Results

### Study Selection

The database search identified 1080 studies. After removing 131 duplicates, 949 studies remained for further consideration. The remaining articles underwent title and abstract analysis, after which 927 studies were excluded, leaving 22 studies for further review. Out of the remaining 22 studies, one was not available due to restricted access to the full text, leaving 21 studies to be screened in full text. However, 12 of these 21 articles were subsequently excluded for the following reasons: Four studies were excluded because they investigated only chronic effects, one of which additionally applied concurrent training protocols. Further, four studies were excluded as they focused solely on myokines (e.g. IL-6 or IL-15) without analyzing additional inflammatory parameters. Another two studies were excluded, one because it involved participants with health impairments, and the other due to its format as an unpublished dissertation. Finally, two studies were excluded due to insufficient data presentation.

**Fig. 1:**
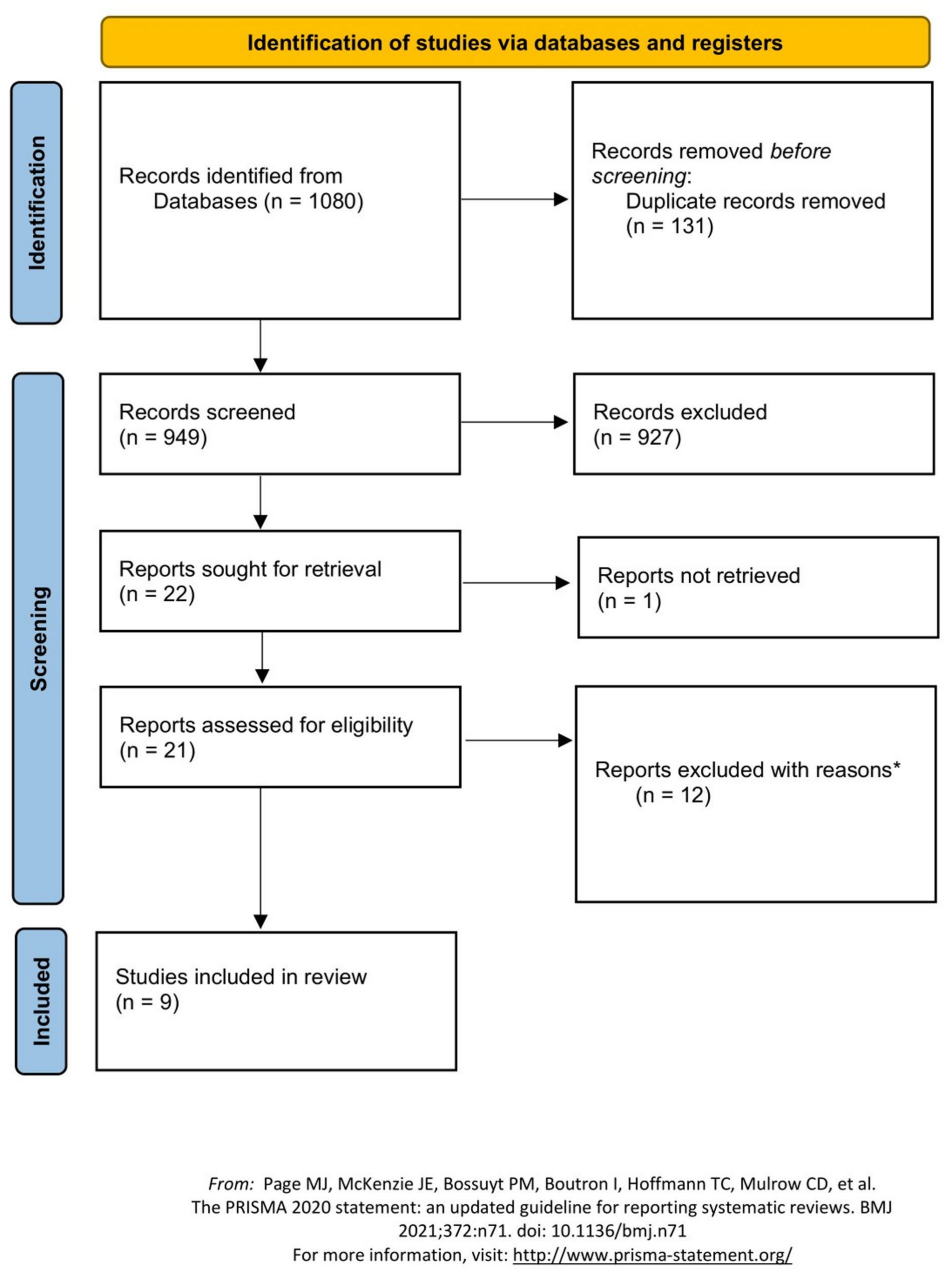
PRISMA 2020 flow diagram. *with reasons = for further information see text

The remaining 9 studies were included in the systematic review. The flow diagram is shown in Fig. 1.

### Study Characteristics

The 9 studies in the systematic review included a total of 189 participants, with a majority of male subjects (156 males, 13 females). No information regarding sex distribution was reported in the study by Bashafaat et al. [48]. The reported participant ages ranged from approximately 16 years [48] to 33 years [49]. While 8 of 9 studies focused on resistance training, the study by Bashafaat et al. [48] investigated BFR combined with interval-based endurance cycling. Notably, Nielsen et al. [50] included two different intervention protocols. The three-week trial included 18 subjects (10 men in BFR, 8 in LL-RT), and the one-week trial also included 18 subjects (10 men in BFR, 8 in HL-RT) [50].

The investigated group size of the selected studies ranged from 6 subjects [51] to 16 subjects [52]. Participants were categorized as either experienced (regular training) [52–54], recreationally trained [50] or physically active [51, 55, 56]. Some studies categorized participants based on physical activity levels (using tools like the IPAQ) [51, 55]. In the study by Bashafaat et al. [48], participants were described as beginner cyclists, no further information on fitness level was provided.

The number of sets for BFR training, HL-RT and LL-RT ranged from 3 [56] to 4 [51–53, 55]. Repetition schemes varied, including 30-15-15-15 [51, 52], repetitions performed to muscular failure [50, 53], and 8, 10, or 25 repetitions per set [56]. Training intensities ranged from 20% of 1RM [50, 52] to 80% of 1RM [53]. In contrast, Shill et al. [54] utilized unilateral isometric exercise with periodic rest intervals.

Contraction types varied across studies, ranging from 1.5 to 5 s for eccentric and concentric phases [50, 51, 53, 55, 56], with one study using isometric contractions lasting up to 5 min [54]. One study did not specify contraction type [52].

BFR cuff pressures varied across studies, ranging from 90 to 197 mmHg [50, 52, 53], while others used 54 to 80% of arterial occlusion pressure (AOP; the pressure required to fully restrict arterial inflow) [51, 54–56]. Further cuff width ranged from 6.35 cm [54] to 26 cm [55]. For further details, refer to Table 1.

An overview of training parameter distributions across the included studies is provided as a violin plot in Additional file 1 (Figure S1).

### Risk of Bias Tools

Risk of bias assessments, following the methodology outlined by Sterne et al. [46, 47], indicate a serious risk of bias within the study conducted by Bjørnsen et al. [52], primarily due to its single-arm design, which substantially limits the ability to account for potential confounders. In addition, the absence of baseline blood measurements limits the interpretability of serum parameters such as creatine kinase and myoglobin [52]. However, inflammatory markers were not affected, as they were assessed only via muscle biopsy [52].

**Fig. 2:**
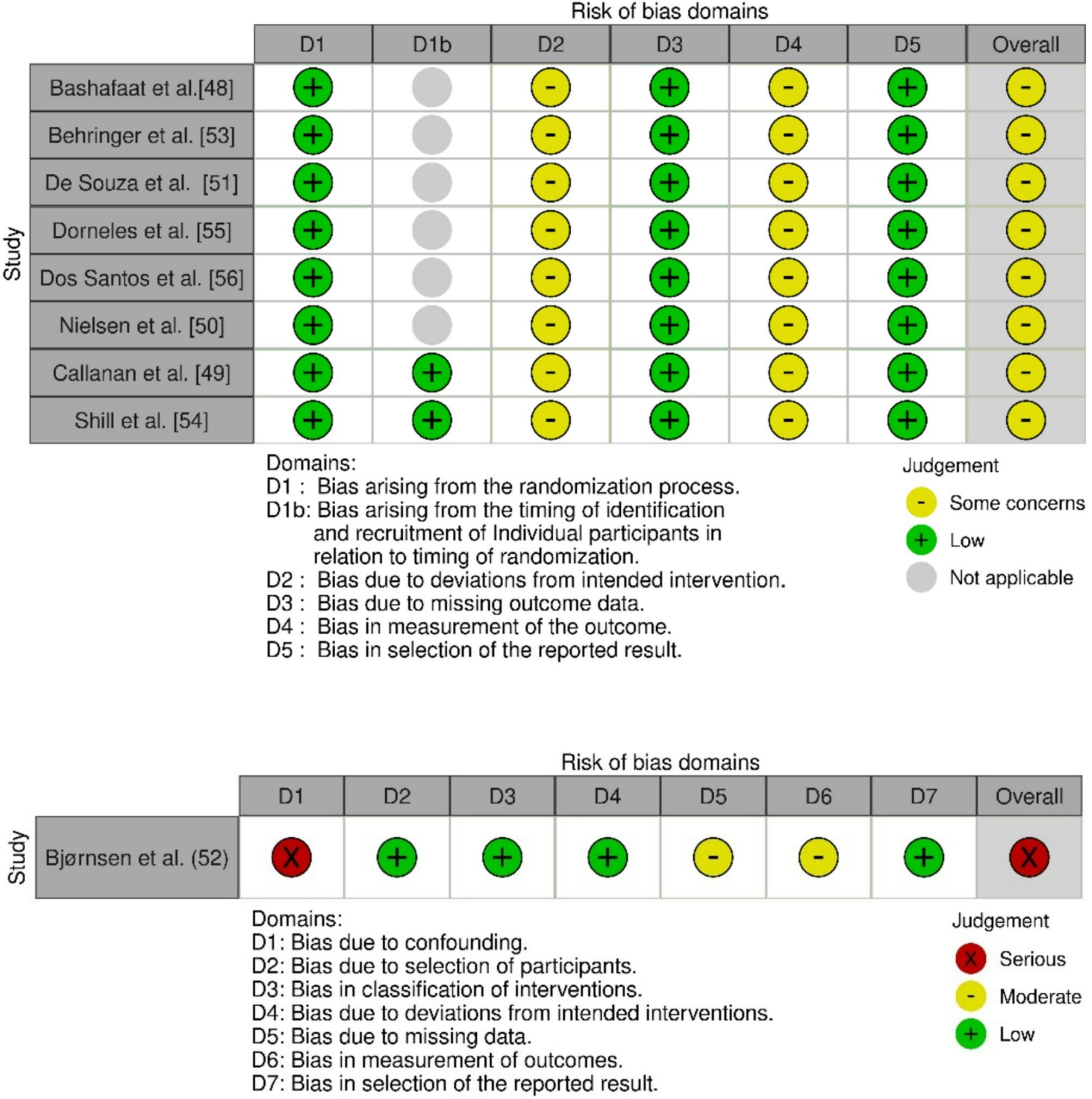
Risk of bias assessment for randomised trials and crossover trials (RoB 2) and for non-randomized studies (ROBIN-I). Domain *D1b* in the RoB 2 tool applies only to crossover trials and was therefore marked as “not applicable” for parallel-group randomized trials

The study by Nielsen et al. [50] was assessed as having some concerns, due to the use of a uniform cuff pressure for all participants. Furthermore, all studies assessed with the RoB 2 tool [48–51, 53–56] were rated as having some concerns, mainly due to the lack of blinding of participants and personnel. The same concern was reflected in the ROBINS-I assessment for the Bjørnsen study [52]. The summarised results are presented in Fig. 2.

### Summary of Effect Directions and Between-Group Comparisons

An overview of the direction and consistency of changes across immune parameters is presented in Table 3.

Heterogeneity was observed regarding the types of inflammatory parameters assessed and the comparator groups applied across studies. Overall, the majority of studies reported increases in inflammatory parameters following BFR training, particularly with regard to total leukocytes, lymphocytes, and macrophages.

In addition, SMD and 95% confidence intervals were calculated for those studies that provided sufficient statistical data (*n* = 4). The resulting SMDs ranged from small to moderate. However, the wide confidence intervals indicate substantial uncertainty (see Table 2). Due to the limited number of studies eligible for effect size calculation, percentage changes remain the primary descriptive metric across this review.

### Effect of BFR Exercise on Immune Parameters

The effect of BFR training on inflammatory parameters was examined in nine studies [50–56], which utilized muscle biopsies [50, 52] and venous blood samples [50, 51, 53–56] to analyze immune parameters. Blood samples were collected immediately after exercise and up to 48 h post-exercise, while muscle biopsies were obtained up to 10 days after the last training session [52]. Overall, the measurement timepoints varied across studies, ranging from immediately post-exercise to several hours or days later (see Table 1). Lymphocytes were analyzed in four studies [51, 55, 56], and leukocytes (neutrophils, monocytes, and macrophages) in five studies [51–53, 56]. Cytokines were investigated in three studies [50, 52, 54], while chemokine-based molecules were additionally identified in two studies [50, 55]. For further details see Tables 2 and 3.

### Leukocytes

An increase in total leukocyte count in peripheral blood was reported in four studies after BFR training (18–38%) [48, 49, 51, 56]. In HL-RT, total leukocyte responses were variable, with both decreases (9%) and increases (33%) reported [51, 56], whereas an increase was observed in LL-RT (14–28%) [49, 51]. Additionally, elevated leukocyte counts (27%) were reported following BFR training combined with endurance exercise [48].

Behringer et al. [53] observed elevated neutrophil counts following BFR (40%) and HL-RT (50%). De Souza et al. [51] and Dos Santos et al. [56] observed either no change or a decrease in peripheral blood neutrophils after BFR training (6%) and HL-RT (12%), while Björnsen et al. [52] observed no changes in muscle tissue. In LL-RT, an increase in blood neutrophils (up to 22%) was observed [49, 51]. Conversely, Callanan et al. [49] reported a transient decrease in neutrophils immediately after BFR training (12%), followed by a gradual increase at 40 min (3%) and 60 min (8%) post-exercise.

Bjørnsen et al. [52] and Nielsen et al. [50], based on muscle biopsies, identified an increase in tissue macrophages after training (200%) (M1: 121–165% and M2: 120–165%). In LL-RT, Nielsen et al. [50] observed an increase in M1 macrophages (108%).

An increase in total lymphocytes in the peripheral blood was observed after BFT training (19–43%) [49, 51, 56] and LL-RT (33–42%) [51, 56]. In contrast, De Souza et al. [51] and Callanan et al. [49] reported no changes following HL-RT and LL-RT, respectively.

After BFR training, increase in TCD4+ (25%) and TCD8 + cells (39%) were also observed [51]. In LL-RT, an increase in TCD8 + cells (28%) was reported, while HL-RT showed decreases or no changes in these cell populations [51]. Further, Dorneles et al. [55] observed a decrease in natural killer cells.

### Cytokines and Inflammation-Associated Factors

Bjørnsen et al. [52] observed increased mRNA expression of pro-inflammatory and anti-inflammatory cytokines, including IL-6 (75%), TNF-α (130%), and IL-1β (340%), as well as elevated IL-4 (60%), within 3 h post-exercise, using muscle biopsies. Nielsen et al. [50] and Shill et al. [54] observed changes in peripheral blood cytokines after BFR training (IL-6: +200%; IL-10: +125%; TNF-α: +150%), with Nielsen et al. [50] additionally noting an increase in the chemokine MCP-1 after BFR (27%) and HL-RT (28%). Following HL-RT, an increase in IL-6 (69%) and decrease in TNF-α (16%) were observed [50]. In addition, Dorneles et al. [55] observed a reduction in the chemokine receptor CCR5 24 h following BFR and HL-RT, while Bjørnsen et al. [52] also reported elevated COX-2 mRNA expression (200%) after BFR.

**Table 1:** Overview of study and intervention characteristics

Author (year)	Fitness level	Sample size and sex	Age	Height and weight	Type of exercise and contraction	BFR cuff pressure and width	Training protocol	Rest time (s)	Duration / training sessions	Measurement timepoints
Bashafaat et al. [48]	Cycling Team NA	BFR-C: 10 students CON-C: 10 students	BFR-C/CON-C: 16.43 ± 1.36 yr	BFR-C/CON-C: 175.46 ± 6.14 cm; 60.07 ± 11.74 kg	BFR-C/CON-C: interval cycling	160 mmHg; 15 cm	BFR-C/CON-C: 10 reps (1 min cycling active (at 8/10 Borg scale) and 1 min recovery (at 4/10 Borg sacle)	BFR-C/CON-C: 1 min	Single bout	*Blood samples*: pre and immediately after exercise
Behringer et al. [53]	Regularly trained and experienced	BFR: 10 men HL-RT: 10 men	BFR/HL-RT: 25.3 ± 3.3 yr	BFR/HL-RT: 185.5 ± 6.5 cm; 81.4 ± 10.0 kg	Knee extension; 2 s eccentric / 1 s concentric	197 ± 33.7 mmHg; 13 cm	BFR; HL-RT: 4 x to muscular failure / 75% 1RM	BFR; HL-RT: 30 s	Single bout	*Blood samples*: pre, immediately after, 20 min, 2 h and 24 h post-exercise
Bjørnsen et al. [52]	Active: last 6 months no strength training of thigh muscles (unspecified)	BFR:12 men and 4 women	BFR: 24 ± 2 yr	BFR: 179 ± 8 cm; 78 ± 12 kg	Knee extension; NA (contraction type)	90–100 mmHg/ 54–64% of AOP; 12 cm	BFR: 30-15-15-15 reps/ 20% 1RM	BFR: 30 s	Two blocks of 7 sessions over 5 days, (10 days of rest between blocks)	*Biopsies*: Baseline, Block 1: Acute 1 (day 1), day 4, rest week (4 days after block 1); Block 2: Acute 2 (day 15) and follow-up (3 days and 14 days after cessation)
Callanan et al. [49]	Tegner score 5.5 ± 1.1 (Tegner Activity Level scale)	BFR/LL-RT: 14 men	BFR/ LL-RT: 30.8 ± 3.7 yr	BFR/ LL-RT: 1.8 ± 0.07 m; 89.6 ± 16.5 kg	seated leg extension, prone hamstring curl and semi-reclined leg press; NA (contraction type)	80% AOP; NA (witdh)	BFR; LL-RT: 30-15-15-15 reps/ 30% 1RM	BFR; LL-RT:30 s	Single bout	*Blood samples*: pre, immediately after, 20 min, 40 min and 60 min post-training
De Souza et al. [51]	Active (IPAQ-based classification)	BFR: 3 men and 3 women LL-RT: 3 men and 3 women HL-RT: 3 men and 3women	BFR: 26.17 ± 3.08 yr LL-RT: 25.20 ± 4.09 yr; HL-RT: 25.20 ± 4.56 yr;	BFR: NA (height); 70.76 ± 12.78 kg LL-RT: NA (height); 65.26 ± 6 6.52 kg HL-RT: NA (height) 72.62 ± 7.41 kg	Bench press and knee extension; 2 s eccentric / 1 s concentric	80% of AOP; 12 cm, 18 cm	BFR; LL-RT: 30-15-15-15 reps/ 30% 1RM HL-RT: 3 × 10 reps/75% 1RM	BFR; LL-RT: 30 s HL-RT: 2 min 15 s	Single bout	*Blood Samples*: pre, immediately after, 30 min, 24 h post-exercise
Dorneles et al. [55]	Physically active- but untrained: last 6 months no strength training (IPAQ)	BFR: 15 men HL-RT: 16 men	BFR: 23.53 ± 2.77 yr HL-RT: 24.46 ± 2.56 yr	BFR: 1,73 ± 0.06 m; 76.73 ± 13.1 kg HL-RT: 1.75 ± 0.05 m; 1.73 ± 0.06 kg	Elbow flexion and knee extension; 2 s eccentric / 1 s concentric	SBP ± 20 mmHg; 14.5 cm; 26 cm	BFR: 4 × 23 reps/ 30% 1RM HL-RT: 4 × 8 reps/ 80% 1RM	BFR; HL-RT: 2 min	Single bout	*Blood Samples*: pre, immediately after and 24 h post- exercise
Dos Santos et al. [56]	Regularly trained and experienced	BFR: 10 men HL-RT: 10 men	BFR: 26.0 ± 6.8 yr HL-RT: 23 ± 5.2	BFR: 168 ± 8 cm; 69.8 ± 12.4 kg HL-RT: 167 ± 10 cm; 66.5 ± 11.5 kg	Leg press; 2 s eccentric / 1 s concentric	80% of AOP; 18 cm	BFR: 3 × 25 reps/ 40% 1RM HL-RT: 3 x to muscular failure/ 80% 1RM	BFR; HL-RT: 1 min	Single bout	*Blood Samples*: pre, immediately after, 24 h and 48 h post- exercise
Nielsen et al. [50]	Recreationally trained: no strength training last 12 months	BFR: 20 men LL-RT: 8 men HL-RT: 8 men	BFR (3w; 1 w trial): 23 ± 2 yr LL-RT (3w trial): 22 ± 2 yr HL-RT (1w trial): 24 ± 3 yr	BFR: *(3w trial)* 181 ± 6 cm; 82 ± 14 kg *(1w trial)* 182 ± 4 cm; 81 ± 9 kg LL-RT: 181 ± 6 cm; 82 ± 14 kg HL-RT: 182 ± 10 cm; 76 ± 4 kg	Knee extension; 1.5 s eccentric/ concentric)	100mmHg; 13.5 cm	BFR; LL-RT: 4 x to muscular failure/ 20% 1RM HL-RT: 4 x to muscular failure/ 70% 1RM	BFR; LL-RT: 30s HL-RT: 90s	3 weeks trial/ 23 sessions & One week trial/ 5 sessions	*Blood Samples*: 5 min pre-training, 5 min, 15 min, 60 min, 180 min, 24 h post first and last session *Biopsies*: pre, 3 and 10 days after cessation
Shill et al. [54]	Vigorous to moderate activity	BFR/ non ischemic: 14 men	BFR/ non ischemic: 21.8 ± 0.4 yr	BFR/ non ischemic: 1.8 ± 0.03 m; 79.1 ± 0.6 kg	forearm handgrip exercise; 5 min isometric hold	95% of SBP; 6.35 cm	BFR; non ischemic: 30 min unilateral intermittent isometric exercise at 65% of MVC	BFR; non ischemic:5 min exercise & 20-s rest	Single bout	*Blood Samples*: 5 min pre-training, 10 min, 30 min, 60 min and 120 min post-training

*min  minute, *h* hour, *w* week, *IPAQ*  International Physical Activity Questionnaire, *BFR-C*  BFR cycling group, *CON-C*  cycling without BFR, *yr*  years, *cm*  centimeter, *m*  meter, *kg*  kilogram, *SBP*  systolic blood pressure at rest, *AOP*  arterial occlusion pressure, *MVC*  maximal voluntary contraction, *d* day, *s* second, *NA* not available, Tegner score 5.5 ± 1.1 = moderately to actively trained

**Table 2:** Overview of inflammatory outcomes following BFR training and control interventions (LL-RT/HL-RT)

Authors	Inflammatory parameters						SMD (95%-KI)
	BFR training			Control group (LL-RT/HL-RT/Cycling)			
Bashafaat et al. [48]	Leukocytes (blood analysis) ↑Total leukocytes from pre to immediately post (≈ 27%)			Leukocytes (blood analysis) CON-C: ↑Total leukocytes from pre to immediately post (≈ 32%)			Leukocytes: 0.47 (− 0.42, 1.36)
Behringer et al. [53]	Leukocytes (blood analysis) ↑ neutrophils immediately post (≈ 40%) and 2 h post cessation (≈ 27%)			Leukocytes (blood analysis) HL-RT: ↑ neutrophils immediately post (≈ 50%) and 2 h post cessation (≈ 28%)			Neutrophils: 0.47, 9 (− 0.78; 1.73)
Bjørnsen et al. [52]	Leukocytes (muscle biopsy per fiber) ↑ tissue macrophages from pre to 1 h post 1st training, to rest week and from 2nd training to 1 h and to 3d and 10 d cessation (≈ 200%); ↔ tissue neutrophils	Cytokines (muscle biopsy) ↑ mRNA of: IL6 (≈ 75%), TNF-α (≈ 130%) from pre-1st training to 1 h post, rest week from 2nd training to 1 h and 3d post; ↑ IL1b (≈ 340%) from 2nd training to 1 h post; ↑ IL4 (≈ 60%) at rest week and from 2nd training to 3 h post; ↑ IL8 (≈ 333%) from pre-1st training to 1 h post and from 2nd training to 1 h and 3d post	Inflammation-Associated Factors (muscle biopsy) ↑ mRNA of COX-2 (≈ 200%) from pre-1st training to 1 h post and from 2nd training to 1 h post cessation	NA			NA
Callanan et al. [49]	Leukocytes (blood analysis) ↑Total leukocytes from pre to immediately post (38%) and ↓ to 40 min (8%) and 60 min post (8%) ↓ neutrophils from pre to immediately post (12%) and ↑ to 40 min (3%) and 60 min post (8%) ↑Total Lymphocytes from pre to immediately post (19%) ↓ to 60 min post (11%)			Leukocytes (blood analysis) LL-RT: ↑Total leukocytes from pre to immediately post (14%) and ↓ to 60 min post (6%) ↑neutrophils from pre to 40 min (4%) and 60 min post (8%) ↔Total Lymphocytes			NA
De Souza et al. [51]	Leukocytes (blood analysis) ↑ total leukocytes to immediately post (≈ 18%) and ↓ to 30 min post (17%); ↓ neutrophils immediately post to 30 min (≈ 6%) and to 24 h post-training (≈ 29%) ↔ monocytes	Lymphocytes (blood analysis) ↑ total lymphocytes from pre until immediately post (≈ 37%); ↓from immediately to post (≈ 36%); and ↑from 30 min to 24 h post-training (≈ 18%) ↑TCD4+ (≈ 25%) and TCD8+ (≈ 39%) from pre to immediately post		Leukocytes (blood analysis) HL-RT: ↓ total leukocytes from immediately to 30 min post (≈ 9%) ↓ neutrophils immediately post to 30 min (≈ 12%) ↔ monocytes LL-RT: ↑ total leukocytes to immediately post (≈ 28%) and ↓ to 30 min post (28%); ↑neutrophils from pre to immediately post (≈ 22%) and ↓ immediately to 30 min post-training (≈ 12%) ↔ monocytes	Lymphocytes (blood analysis) HL-RT: ↔ total lymphocytes ↓TCD4 + from immediately post to 30 min post (≈ 1 6%) ↔ TCD8+ LL-RT: ↑ total lymphocytes from pre until immediately post (≈ 42%); ↓from immediately to 30 min post (≈ 35%) ↓TCD4 + from immediately post to 30 min post (≈ 15%) ↑ TCD8+ (≈ 28%) from pre to immediately post		NA
Dorneles et al. [55]		Lymphocytes (blood analysis) ↔ natural killer cells from pre- to post-training	Inflammation-Associated Factors (blood analysis) ↓ CCr5 from immediately post to 24 h post-training (≈ 29%)	Lymphocytes (blood analysis) HL-RT: ↓ natural killer cells from pre- to 24 h post-training (≈ 39%)	Inflammation-Associated Factors (blood analysis) HL-RT: ↓ CCr5 from pre to 24 h post-training (≈ 22%)		NA
Dos Santos et al. [56]	Leukocytes (blood analysis) ↑ total leukocytes from pre to immediately post-training (33%) ↔ neutrophils and monocytes	Lymphocytes (blood analysis) ↑ total lymphocytes from pre to immediately post-training (43%)		Leukocytes (blood analysis) HL-RT: ↑ total leukocytes from pre to immediately post-training (33%) ↔ neutrophils and monocytes	Lymphocytes (blood analysis) HL-RT: ↑ total lymphocytes from pre to immediately post-training (33%) ↔ neutrophils and monocytes		Leukocytes: 0.91 (–0.01; 1.83) Lymphocytes: 0.60 (-0.30; 1.50)
Nielsen et al. [50]	Leukocytes (3 weeks trial) (muscle biopsy) per mm² (fiber cross-sectional area): ↑ MPs (M1) and MPs (M2) from pre to 3d post cessation (up to 121%); ↑ M2 (up to 120%) from pre to 10 d post cessation; MPs expressed per 100 myofibers: ↑ M1 (165%) and M2 (163%) from pre to 3d after cessation	Cytokines (One week trial) (blood analysis) ↔ IL-6 from pre to first session and ↓ from pre to 180 min post (18%); ↔ TNF-α	Inflammation-Associated Factor(One week trial) (blood analysis) ↑MCP-1 from pre at last training session (33%)	Leukocytes (3 weeks trial) (muscle biopsy) per mm² (fiber cross-sectional area) LL-RT: ↑ MPs (M1) from pre to 3d post cessation (up to 107%) MPs expressed per 100 myofibers: ↑ M1 (108%) from pre to 3d post cessation	Cytokines (One week trial) (blood analysis) HL-RT: ↑L-6 from pre to first session (up to 69%) ↓TNF-α from pre to 24 h post (up to 16%)	Inflammation-Associated Factors(One week trial) (blood analysis) HL-RT: ↑*MCP-1 from pre at first training session (28%)	M1-macrophages (fiber cross-sectional area): 0.16 (− 0.77; 1.10) M1-macrophages (expressed per 100 myofibers): − 0.10 (-1.03; 0.83) MCP: NA Cytokines: NA
Shill et al. [54]		Cytokines (blood analysis) ↑TNF-α (≈ 150%), IL-6 (≈ 200%), IL-10 (≈ 125%) from pre to 30 min post-training		Cytokines (blood analysis) Control group (non-ischemic): ↓ from TNF-α (≈ 31%), IL-6 (≈ 28%) from pre to 30 min post-training			NA

*↑= increase, ↓= decrease, ↔= no changes, *mm*  millimetre, *SBP*  systolic blood pressure at rest, *AOP*  arterial occlusion pressure, *MVC*  maximal voluntary contraction, *RF*  rectus femoris, *VM*  vastus medialis, *VIM*  vastus intermedius, *d*  day, *CCr5*  chemokine receptor type 5, *MP*  macrophage, *MCP-1* monocyte chemotactic protein 1, *s*  second, *%* percentages calculated on the basis of graphical data from original studies, *NA*  not available, ↑*=tendency to increase; *Con-C * cycling without BFR; *SMD*   standardized mean difference

**Table 3:** Effect direction plot: summary of the direction of change in inflammatory parameters across included studies

Authors	Study Design	Intervention Group	Total Leuko cytes	Total Lympho cytes	(TCD4+/ TCD8+)		Natural killer cells	Neutro phils	Mono cytes	Macrophags (M1 / M2)		Macrophages	IL-6	TNF-α	IL1b	IL4	IL8	IL10	MCP-1	COX- 2	CCr5
Bashafaat et al. [48]	RCT	BFR-C	▲																		
		CON-C	▲																		
Behringer et al. [53]	RCT	BFR						▲													
		HL-RT						▲													
Bjørnsen et al. [52]	SAT	BFR						◆				▲	▲	▲	▲	▲	▲			▲	
		NA																			
Callanan et al. [49]	RCOT	BFR	▲	▲				▲													
		LL-RT	▲	◆				▲													
De Souza et al. [51]	RCT	BFR	▲	▲	▲	▲		▼	◆												
		LL-RT	▲	▲	▼	▲		▲	◆												
		HL-RT	▼	◆	▼	◆		▼	◆												
Dorneles et al. [55]	RCT	BFR					◆														▼
		HL-RT					▼														▼
Dos Santos et al. [56]	RCT	BFR	▲	▲				◆	◆												
		HL-RT	▲	▲				◆	◆												
Nielsen et al. [50]		BFR								▲	▲		◆	◆					▲		
	RCT	LL-RT								▲	◆								▲*		
		HL-RT											▲	▼							
Shill et al. [54]	RCOT	BFR											▲	▲							
		CG											▼	▼							

*▲= significant increase (*P* < 0.05); ▼= significant decrease (*P* < 0.05); ◆= no changes (*P* ≥ 0,05); ▲*= tendency to increase; *CG* = Control Group (non ischemic), *BFR-C* = BFR cycling group, *CON-C* = cycling without BFR, *NA* = not available, *CCr5* = chemokine receptor type 5, *MCP-1* = monocyte chemotactic protein 1, *COX-2* = Cyclooxygenase-2, *RCT* = Randomized Controlled Trial, *RCOT* = Randomized Crossover Trial, *SAT* = Single-Arm Trial

## Discussion

**Fig. 3:**
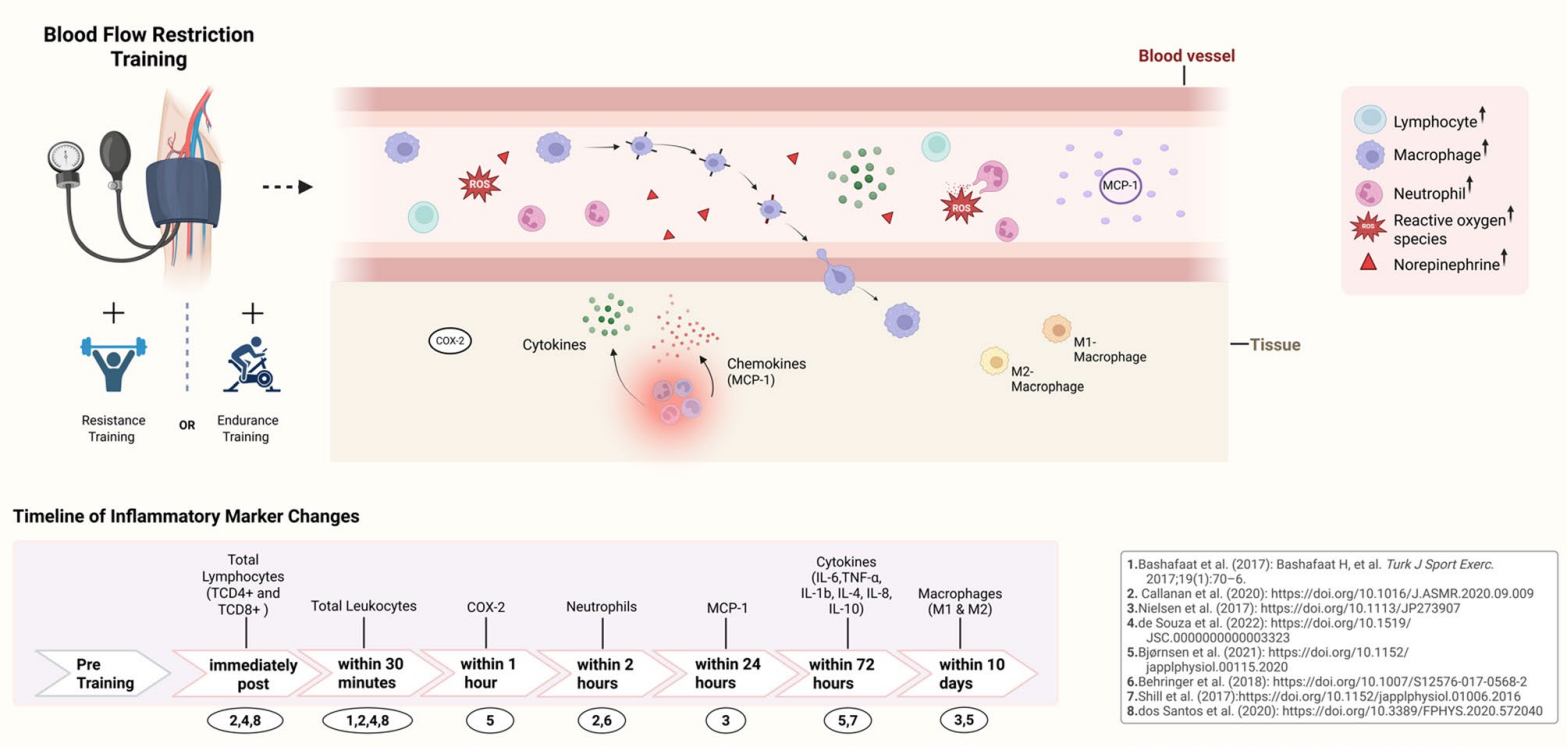
Potential acute inflammatory process following BFR training, evidenced by changes in circulating inflammatory parameters. Created with BioRender.com

This systematic review aimed to examine whether BFR training induces acute inflammation by analyzing changes in inflammatory parameters. The findings of the review suggest that BFR training induces an acute inflammatory response, characterized by transient increases in circulating total leukocytes and lymphocytes, alongside enhanced macrophage activity within the tissue. The responses were observed after both resistance and endurance BFR training and occurred within the first 48 h following BFR training, with tissue macrophages remaining elevated for up to 10 days (see Fig. 3).

### Leukocyte Mobilization Following BFR Training

Previous studies have shown that leukocytes respond rapidly to acute exercise, contributing significantly to exercise-induced leukocytosis [10]. While leukocytosis typically persists during the recovery phase, it has been shown to decrease within approximately 30 min following prolonged or intense exercise [10]. Several studies have consistently demonstrated transient leukocytosis and lymphocytosis immediately after BFR training, with leukocyte counts increasing by around 18–38% and lymphocyte counts by 19–43%, followed by a decline after about 30 min [51, 56]. These transient changes are similar to the immune responses observed in LL-RT and HL-RT control groups within the same studies. Specifically, leukocyte counts increased by up to 33%, while lymphocyte counts were elevated by up to 50% in both LL-RT and HL-RT [51, 56]. In comparison, eccentric exercise involving high mechanical loads has been associated with even more pronounced leukocytosis, with one study reporting a 49% increase in leukocyte counts [9, 11]. This pronounced increase in leukocyte counts following eccentric exercise may be attributed to the substantial muscle damage typically induced by eccentric contractions, which promote local inflammatory signaling and subsequent leukocyte recruitment [9, 11, 22].

### Neutrophil Responses Following BFR Training

In addition to changes in total leukocytes and lymphocytes, some studies have also reported alterations in neutrophils and tissue macrophages following BFR training, whereas monocytes remained largely unaffected [51–53, 56]. It has been shown that the neutrophil response following BFR training (+ 40%) is comparable to that observed in the HL-RT control group (+ 50%) [53]. This aligns with a systematic review that demonstrated a 51% increase in neutrophils following endurance and HL-RT [57].

Nevertheless, neutrophil responses to BFR training varied across the included studies [51–53, 56]. While Behringer et al. [53] observed an increase in peripheral blood neutrophil counts at an intensity of 75% of 1RM, Bjørnsen et al. [52], De Souza et al. [51] and Dos Santos et al. [56] reported either a decrease or no significant change in tissue or peripheral blood neutrophil counts with training intensities ranging from 20 to 40% of 1RM. The differences in neutrophil counts between Bjørnsen et al. [52] and Behringer et al. [53] may be attributed to methodological differences. Bjørnsen et al. [52] used muscle biopsies to assess neutrophil infiltration in the tissue, whereas Behringer et al. [53] measured circulating neutrophil counts in blood samples. Since local tissue biopsies and peripheral blood analyses capture different aspects of inflammation, variations in immune cell distribution between tissue and circulation may account for the divergent findings. Additionally, discrepancies in peripheral blood measurements may be attributed to variations in exercise protocols, particularly training intensity and contraction type. Notably, Behringer et al. [53] combined BFR training with eccentric exercise at 75% of 1RM. Eccentric contractions, especially under high-load conditions, are known to induce greater EIMD than isometric or concentric contractions [9, 11, 13]. EIMD is believed to trigger localized inflammatory responses, as damaged muscle fibers release signals (e.g. chemokine) that recruit immune cells, such as neutrophils to the injury site [11, 20, 58]. This aligns with the findings of Behringer et al. [53], who reported EIMD along with an increase in neutrophil counts. Moreover, metabolic stress due to reduced oxygen supply during eccentric contractions has been proposed as a contributing factor to membrane damage [53]. This damage has been associated with ROS production during ischemia-reperfusion, which may lead to structural disruptions and an amplified inflammatory response [34, 35, 59]. Neutrophils may further contribute to this process, as they are known to produce ROS themselves [10, 18, 35]. In summary, the variability in neutrophil responses across studies is likely driven by differences in exercise protocols, particularly in terms of mechanical and metabolic stress.

### Role of Catecholamines in Leukocyte Recruitment

While most studies reported no or minimal muscle damage following BFR training [25, 31, 32], alternative mechanisms may be responsible for the recruitment of leukocytes [48–52, 55, 56]. Catecholamines such as norepinephrine appear to play a key role in this process [18]. Previous studies have shown that norepinephrine facilitates the detachment of leukocytes from the endothelium, thereby increasing their concentration in the bloodstream [60, 61]. Accordingly, the observed increase in norepinephrine following BFR training could contribute to leukocytosis [8]. In this context, the rise in catecholamines appears to contribute to the transient lymphocytosis immediately following BFR training [51, 55, 56]. Since lymphocytes possess a high density of β2-adrenoceptors on their cell surface, they may be particularly sensitive to the increase in catecholamines, which could mobilize them from the marginated pool into the circulation [62].

In line with this, increases in total leukocyte counts were also observed following BFR training combined with endurance cycling [48]. This leukocytosis may be attributed to mechanisms typically associated with endurance exercise, such as elevated cardiac output, shear stress, and catecholamine release, which are known to promote the demargination of leukocytes from the endothelial wall [10, 60].

Given that the extent of catecholamine release during exercise is linked to the individual training status, this may also impact the inflammatory responses [63].

### Macrophage Infiltration and Function Following High-Frequency BFR Training

Macrophages are key mediators of the inflammatory response and play a crucial role in tissue repair and regeneration following muscle injury [19, 64]. As the predominant leukocytes in injured muscle, macrophages are suggested to play a key role in muscle recovery [20]. Accordingly, Bjørnsen et al. [52] observed changes in tissue macrophages associated with reported EIMD. However, the interpretation of these findings remains limited due to the absence of baseline measurements for muscle damage markers.

In contrast, Nielsen et al. [50] reported an increase in tissue macrophages (M1 and M2) following high-frequency BFR training, even in the absence of significant EIMD. It has been hypothesized that repeated BFR sessions over several days may lead to cumulative metabolic stress, potentially causing minor myocellular damage, as indicated by localized membrane stress markers [50]. The associated increase in ROS production could compromise the structural integrity of the muscle membrane, thereby triggering a localized inflammatory response [34], which may explain the observed macrophage infiltration.

Further, Nielsen et al. [50] specifically observed an increase in both pro-inflammatory (M1) and anti-inflammatory (M2) macrophages, with a predominance of M2 macrophages, which are associated with tissue regeneration [18–20]. In this context, the increase in M1 macrophages (108–165%) was similar to that seen in the LL-RT control group, whereas an increase in M2 macrophages (163%) was observed only following BFR training.

Moreover, previous studies have demonstrated that macrophages can be polarized into M1 and M2 phenotypes, depending on the microenvironment and inflammatory signals [18–20]. M1 macrophages are considered to be involved in the early stages of inflammation, promoting pro-inflammatory signaling, while M2 macrophages are thought to play a role in resolving inflammation and supporting tissue regeneration [18, 19]. The limited presence of M1 macrophages observed by Nielsen et al. [50] likely indicates minor myocellular stress, while the predominance of M2 macrophages suggests a shift toward inflammation resolution and tissue remodeling. This pattern of macrophage activation may reflect a localized inflammatory response that supports muscle regeneration following BFR training. This suggests an association between metabolic stress and the inflammatory response, which may in turn contribute to muscle adaptation through macrophage recruitment and polarization.

#### Cytokine Response Following BFR Training

Cytokines are signaling molecules that regulate immune function and play a crucial role in maintaining tissue homeostasis [65]. The balance between pro-inflammatory and anti-inflammatory cytokines is essential for controlling inflammation [65]. Several pro-inflammatory cytokines (IL-6, IL-8, IL-1b, and TNF-α) and anti-inflammatory cytokines (IL-4, IL-10) have been observed following BFR training [10, 66]. However, results from the included studies show discrepancies between cytokine concentrations in peripheral blood and their mRNA expression in tissue, particularly for IL-6 and TNF-α [52, 54, 66]. These discrepancies may arise because cytokines are secreted by various cell types and tissues, with muscle cells being a major contributor during exercise [67]. Consequently, circulating cytokines may not accurately reflect tissue concentrations.

Furthermore, cytokine production appears to be closely linked to immune cell activity. Increased cytokines have been observed in the presence of immune cells, particularly macrophages [68, 69]. Experimental studies in animal models demonstrate that macrophages stimulate the expression of cytokines such as IL-6 and TNF-α [68–70], suggesting that macrophage infiltration into muscle tissue following BFR training may contribute to cytokine production.

In addition to immune cell activation, hormonal factors such as catecholamines have been shown to enhance cytokine production, particularly IL-6 [71]. Notably, IL-6 has been identified as the most prominent and consistent exercise-induced cytokine response [10]. This pleiotropic cytokine has been proposed to have a dual role, acting as both an inflammatory marker and an anabolic signal [72, 73]. IL-6 is also thought to stimulate anti-inflammatory cytokines and facilitate immune cell recruitment [72, 74–76]. For instance, a study in mice demonstrated that blocking the IL-6 receptor led to a decrease in neutrophil numbers [75], indicating that IL-6 may regulate the inflammatory response by influencing immune cell mobilization.

Moreover, IL-6 expression has been reported to be regulated via ROS-dependent signaling pathways [77]. Given that ROS are produced during the metabolic response to exercise, IL-6 may represent a link between exercise-induced stress and inflammatory signaling, especially under BFR-induced ischemia-reperfusion conditions [77–79]. In line with this, IL-6 plasma concentrations following BFR have been shown to increase by up to 200%, which appears comparable to or even greater than responses reported for HL-RT and endurance exercise (up to 145%), suggesting a potentially comparable role in exercise-induced inflammation [57].

Overall, the biological functions of many exercise-induced cytokines remain poorly understood, and their isolated measurement may not provide a comprehensive understanding of the inflammatory response. This highlights the need for further research to clarify their specific roles in exercise-induced inflammation.

### Inflammation-Associated Factors Following BFR Training

COX-2, a key enzyme involved in mediating inflammation and pain, has been observed following BFR training in one of the analyzed studies [52]. Although PGE-2 was not directly measured in that study, the presence of COX-2, which catalyzes the production of PGE-2, suggests that similar inflammatory pathways may be activated [75, 76]. Supporting this notion, several studies have shown that the progressive increase in COX-2 expression correlates with elevated PGE-2 concentration [81, 82]. Further evidence is provided by studies on non-steroidal anti-inflammatory drugs (NSAIDs), which have been shown to reduce inflammatory parameters by inhibiting COX-2 activity [80, 82, 83]. This inhibition decreases the synthesis of PGE-2, thereby reducing the overall inflammatory response [82]. Thus, the observed presence of COX-2 may indicate its role in mediating local inflammatory pathways following BFR training.

In addition, increased peripheral blood MCP-1 concentration was reported by Nielsen et al. [50] following BFR training. MCP-1 is a potent chemotactic and activating factor and is believed to play a crucial role in recruiting macrophages to sites of inflammation [19, 84]. This aligns with findings from animal studies showing that MCP-1 is essential for macrophage recruitment [19, 85]. The concurrent increase in MCP-1 and macrophages observed by Nielsen et al. [50] suggests that MCP-1 likely contributed to the recruitment of macrophages during BFR training.

Taken together, these findings underscore the presence of an acute inflammatory milieu following BFR training, which is thought to contribute to muscle regeneration. This assumption is particularly supported by the observed increase in macrophages (especially M2) and neutrophils [50, 53], as these cell types are believed to play a crucial role in coordinating muscle repair and adaptation processes [11, 86, 87].

#### Limitations

Some limitations of the present review should be carefully considered. An important limitation is the heterogeneous study methodologies, particularly in terms of training protocols (e.g., exercise intensity, contraction type, cuff pressure and width, single versus multiple sessions), the inflammatory parameters measured, and the measurement intervals. Regarding measurement approaches, some studies analyzed local inflammatory parameters using muscle biopsies, while others focus on peripheral blood samples. In studies involving muscle biopsies, results may also be influenced by the selection of specific muscle regions, as inflammatory responses can vary across different muscle areas. Another limitation is that Bjørnsen et al. [52] investigated only one group without a control group, which limits the ability to rule out alternative explanations for the observed effects. Collectively, these methodological differences complicate direct comparisons between studies and may impact the interpretation of inflammatory responses following BFR training.

Moreover, the low proportion of female participants (approximately 8%) represents an important limitation that should be considered in future research, given that hormonal fluctuations across the menstrual cycle, particularly estrogen levels, may modulate inflammatory responses [88]. In addition, most included studies investigated physically active or trained individuals, while no data are available for sedentary populations, which limit the generalizability of the findings.

Since the primary aim of this review was to determine whether BFR training induces an acute inflammatory response, future research should aim to standardize these variables to enhance comparability and reliability of findings.

## Conclusion

This systematic review indicates that BFR training induces acute inflammation, as evidenced by changes in inflammatory parameters observed following both resistance and endurance exercise. Observed responses include transient increases in total leukocytes and lymphocytes, as well as macrophage infiltration persisting for up to 10 days. Findings on neutrophil and cytokine responses were inconsistent across studies. The discrepancies in immune cell populations may have occurred due to various factors (e.g., methodological factors).

Although direct comparisons with LL-RT remain limited, the magnitude of immune responses following BFR training appears similar to that observed after HL-RT, particularly in terms of total leukocyte and lymphocyte counts, as well as neutrophil mobilization. Given that inflammation following HL-RT has been associated with muscle remodeling, the comparable inflammatory pattern observed after BFR training may likewise indicate a role for inflammation in adaptive processes.

However, while EIMD following HL-RT is associated with inflammatory responses, its role in BFR training remains unclear. Instead, changes in inflammatory markers may be driven by factors such as metabolic stress and hormonal responses. Since the underlying mechanisms remain poorly understood, future research should aim to investigate the pathways involved to clarify the initiation of the inflammatory response and its potential role in muscle regeneration. This knowledge is crucial to optimize recovery strategies, support adaptive processes, and contribute to the safe application of BFR training in both athletic and rehabilitative contexts.

## Supplementary Information

Below is the link to the electronic supplementary material.

Supplementary Material 1

## Data Availability

All analysed data are presented in the main article or in the supplementary material (Additional File 1), which includes additional visualizations.

## Abbreviations

BFR: Blood flow restriction
LL-RT: Low-load resistance training
HL-RT: High-load resistance training
RCT: Randomized controlled trial
RCOT: Randomized crossover trial
SAT: Single-arm trial
EIMD: Exercise-induced muscle damage
ROS: Reactive oxygen species
1RM: One repetition maximum
IPAQ: International physical activity questionnaire
AOP: Arterial occlusion pressure
ROB 2: Risk-of-bias tool for randomized trials 2
ROBIN-I: Risk of bias in non-randomized studies of interventions
SMD: Standardized mean difference
SWiM: Synthesis without meta-analysis
NA: Not available
