# Supplementary material for: Acute Inflammatory Responses to Blood Flow Restriction Training: A Systematic Review
Source: Sports Med Open. 2025 Oct 21;11:121. doi: 10.1186/s40798-025-00926-6 (PMC12540228; doi:10.1186/s40798-025-00926-6)
Supplement: Supplementary file 1 — Supplementary Material 1 [file 40798_2025_926_MOESM1_ESM.docx]

*Supplementary Information for publication in*
**Sports Medicine – Open**

# **Acute Inflammatory Responses to Blood Flow Restriction Training: A Systematic Review**

Sarah Barawi^1^, Kevin Happ^1^, Michael Behringer^1^

^1^Department of Sports Medicine and Exercise Physiology, Institute of Sports Sciences, Goethe University Frankfurt, Frankfurt am Main, Germany.

**Corresponding author**

Sarah Barawi

Goethe University Frankfurt

Department of Sports Sciences

Ginnheimer Landstraße 39, D-60487 Frankfurt am Main, Germany

Email: [barawi@sport.uni-frankfurt.de](mailto:barawi@sport.uni-frankfurt.de)

**Email (co-authors)**

[happ@sport.uni-frankfurt.de](mailto:happ@sport.uni-frankfurt.de)

[behringer@sport.uni-frankfurt.de](mailto:behringer@sport.uni-frankfurt.de)

**Literature Search Strategy**

**Table S1.** Search strategy

| **Database** | **Search terms** |
| --- | --- |
| **Pubmed** | ("BFR training"[tw] OR "blood flow restriction training"[tw] OR "KAATSU training"[tw] OR "occlusion training"[tw] OR intermittent ischemic exercise[tw]) AND (inflammat*[tw] OR "inflammatory response"[tw] OR "inflammatory reaction"[tw] OR "immune response"[tw] OR "inflammatory marker*"[tw] OR "inflammatory parameter*"[tw] OR "inflammation mediator*"[tw] OR "immune marker*"[tw] OR cytokine*[tw] OR interleukin*[tw] OR leukocyt*[tw] OR "white blood cell*"[tw] OR lymphocyt*[tw] OR monocyt*[tw] OR neutrophil*[tw] OR macrophage*[tw] OR "cytokines"[MeSH] OR "leukocytes"[MeSH] OR "Lymphocytes"[MeSH] OR "Monocytes"[MeSH] OR "neutrophil"[MeSH] OR "macrophages"[MeSH]) |
| **Web of Science** | ("BFR training" OR "blood flow restriction training" OR "KAATSU training" OR "occlusion training" OR "intermittent ischemic exercise") AND (inflammat* OR "inflammatory response" OR "inflammatory reaction" OR "acute inflammation" OR "immune response" OR "immunological response" OR "inflammatory marker*" OR "inflammatory parameter*" OR "immune marker*" OR cytokine* OR interleukin* OR leukocyt* OR "white blood cell*" OR lymphocyt* OR monocyt* OR neutrophil* OR macrophage*) |
| **Google Scholar and BISp** | ("BFR training" OR "blood flow restriction training" OR "KAATSU training" OR "occlusion training" OR "intermittent ischemic exercise") AND ("inflammatory response" OR "immune response" OR "inflammatory marker" OR "immunological response" OR "cytokine" OR "interleukin" OR "leukocytes" OR "white blood cells" OR "lymphocytes" OR "monocytes" OR "neutrophils" OR "macrophages") |

**Violin Plots of Training Parameters
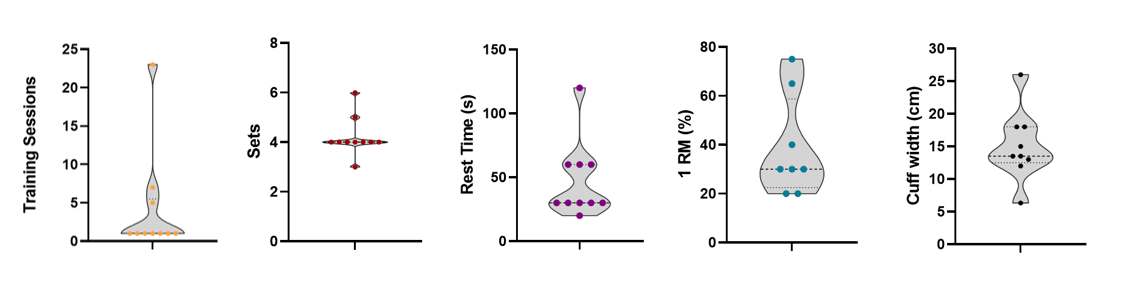
 Figure S1.** Violin plots of training parameters reported in the included studies. Each dot represents one study. Nielsen et al. [50] conducted two separate trials, resulting in two dots. Bashafaat et al. [48] conducted an endurance-based BFR study, hence no data point is shown in the 1RM plot.

**Reasons for Excluded Studies**

| **Author** | **Reason for Exclusion** |
| --- | --- |
| Karabulat et al. (1) | Solely investigated IL-6 as, without assessing additional immune parameters |
| De Lima et al. (2) | Focused exclusively on long-term training effects |
| Kazemi et al. (3) | Measured IL-15 only, classified as a myokine rather than a classical inflammatory marker |
| Nakajima et al. (4) | Included participants with pre-existing medical conditions |
| Eslamdoust et al. (5) | Solely investigated IL-6 as inflammatory marker |
| Jahandarlashaki et al. (6) | Focused exclusively on long-term training effects |
| Poole (7) | Dissertation |
| Asadi et al. (8) | Reported incomplete/ insufficient data for extraction |
| Winchester et al. (9) | Solely investigated IL-6 as inflammatory marker |
| De Souza et al. (10) | Focused exclusively on long-term training effects and concurrent training |
| Qian et al. (11) | Reported incomplete/ insufficient data for extraction |
| Laswati et al. (12) | Focused exclusively on long-term training effects |

**References**

1. Karabulut M, Sherk VD, Bemben DA, Bemben MG. Inflammation marker, damage marker and anabolic hormone responses to resistance training with vascular restriction in older males. Clin Physiol Funct Imaging. 2013 Sep;33(5):393-9. doi: 10.1111/cpf.12044. Epub 2013 Apr 23. PMID: 23701309.
2. de Lima FR, Marin DP, Ferreira LT, Sousa Filho CPB, Astorino TA, Prestes J, Marquezi ML, Otton R. Effect of Resistance Training With Total and Partial Blood Flow Restriction on Biomarkers of Oxidative Stress and Apoptosis in Untrained Men. Front Physiol. 2021 Sep 9;12:720773. doi: 10.3389/fphys.2021.720773. PMID: 34566685; PMCID: PMC8458755.
3. Kazemi, Abdolreza, and Behroz Vakilzadeh. "The effects of resistance training with BFR on serum IL-15 and folestatin in young male athletes." *Sport Physiology & Management Investigations* 13.3 (2021): 159-170.
4. Nakajima, T., et al. "The effects of low-intensity KAATSU resistance exercise on intracellular neutrophil PTX3 and MPO." *International Journal of KAATSU Training Research* 8.1 (2012): 1-8.
5. Eslamdoust, Mohammad, et al. "The Effect of Eccentric Resistance Training with and Without Blood Flow Restriction on Serum IL6 and MMP9 Levels in Young Active Men." *Journal of Archives in Military Medicine* 7.4 (2019).
6. Jahandarlashaki, T., et al. "Long-term effects of resistance exercise with and without vascular occlusion on TNF-α, IL-6 and IL-15 secretion in non-athletic women." *Comparative Exercise Physiology* 19.4 (2023): 323-330.
7. Poole, Christopher. *An evaluation of the inflammatory time course response following traditional and blood flow restriction resistance exercise measured by peripheral quantitative computed tomography*. The University of Oklahoma, 2012.
8. Asadi, Mohammad Bani, et al. "Acute inflammatory response to a single bout of resistance exercise with or without blood flow restriction." *International Journal of Sport Studies for Health* 3.2 (2020): e110594.
9. Winchester, Lee J.1; Morris, Cody E.2; Badinger, Joseph3; Wiczynski, Teresa L.3; VanWye, William R.4. Blood Flow Restriction at High Resistance Loads Increases the Rate of Muscular Fatigue, but Does Not Increase Plasma Markers of Myotrauma or Inflammation. Journal of Strength and Conditioning Research 34(9):p 2419-2426, September 2020. | DOI: 10.1519/JSC.0000000000003742
10. de Souza TMF, Libardi CA, Cavaglieri CR, Gáspari AF, Brunelli DT, de Souza GV, Ugrinowitsch C, Min Li L, Chacon-Mikahil MPT. Concurrent Training with Blood Flow Restriction does not Decrease Inflammatory Markers. Int J Sports Med. 2018 Jan;39(1):29-36. doi: 10.1055/s-0043-119222. Epub 2017 Nov 9. PMID: 29121681.
11. Qian, H., Shu, W., Wen, S. *et al.* Effects of LICT-BFR on cardiopulmonary fitness and immune function in youth. *Sport Sci Health* **20**, 1235–1242 (2024). <https://doi.org/10.1007/s11332-024-01195-8>
12. Laswati H, Sugiarto D, Poerwandari D, Pangkahila JA, Kimura H. Low-Intensity Exercise with Blood Flow Restriction Increases Muscle Strength without Altering hsCRP and Fibrinogen Levels in Healthy Subjects. Chin J Physiol. 2018 Jun;61(3):188-195. doi: 10.4077/CJP.2018.BAG567. PMID: 29962179.
